# Supplementary figures and images for: Root remodeling mechanisms and salt tolerance trade-offs: The roles of HKT1, TMAC2, and TIP2;2 in Arabidopsis
Source: PLoS Genet. 2025 Jun 11;21(6):e1011713. doi: 10.1371/journal.pgen.1011713 (PMC12204623; doi:10.1371/journal.pgen.1011713)

**A**

# UAS-HKT1 vs. background

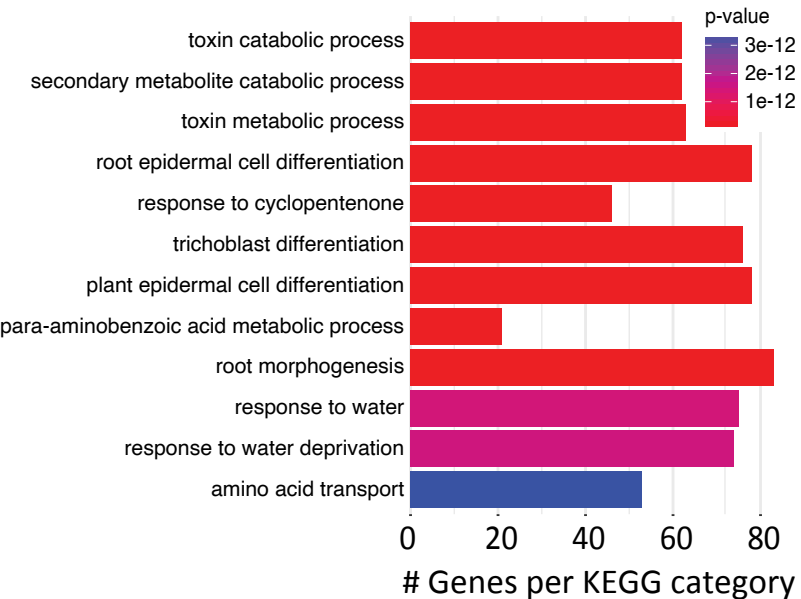**B**

# 0 mM NaCl vs 75 mM NaCl

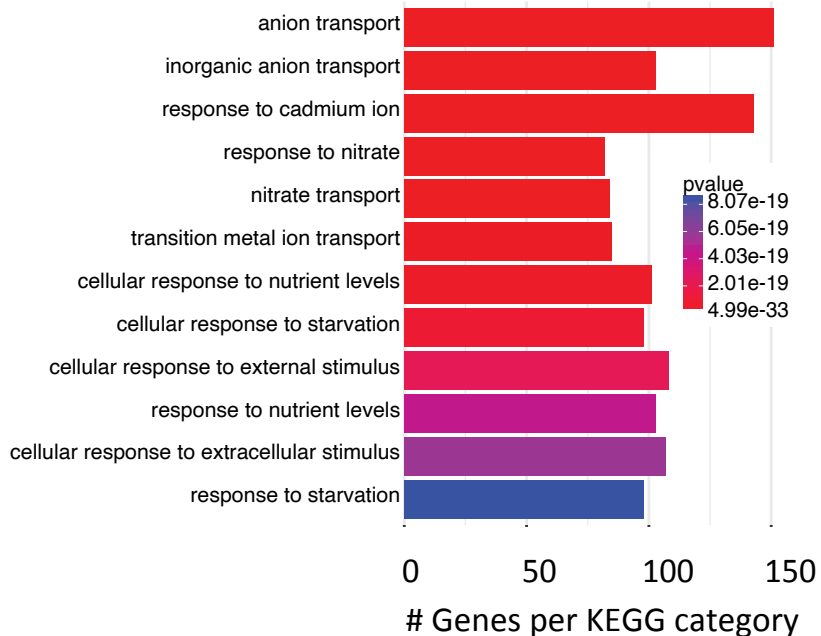**C**

# 0 mM NaCl vs 30 mM KCl + 75 mM NaCl

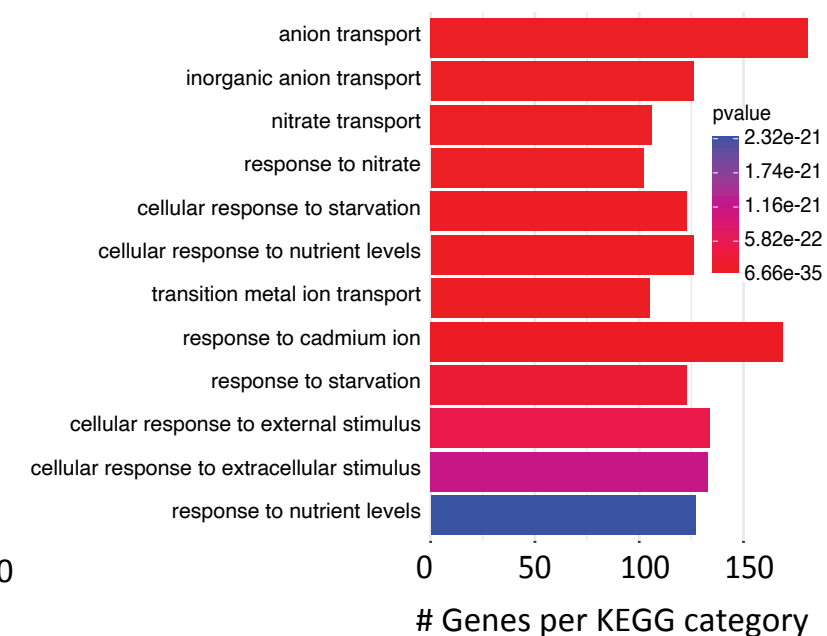

Supplement: S1 Fig — The abundance of differentially expressed genes was evaluated per KEGG category for pair-wise comparisons (A) UASGAL4:HKT1 versus background; (B) control 0 mM versus stress at 75 mM NaCl and (C) control 0 mM NaCl versus supplemental K+ (30 mM KCl) during salt stress (75 mM NaCl). (PDF) [file pgen.1011713.s001.pdf]

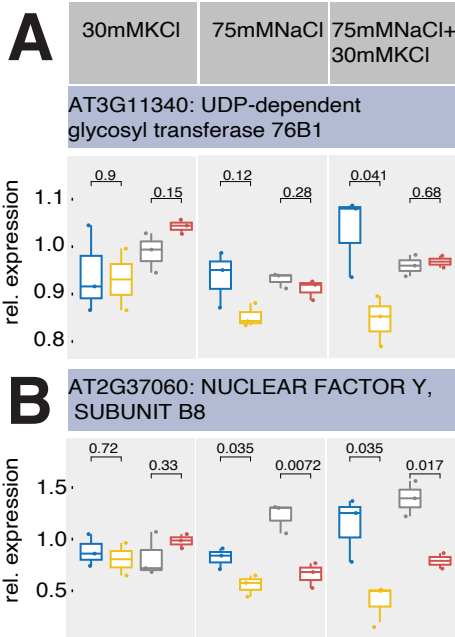

Genotype

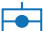 E2586 
 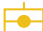 E2586 UAS-HKT1  
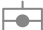 J2731 
 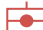 J2731 UAS-HKT1

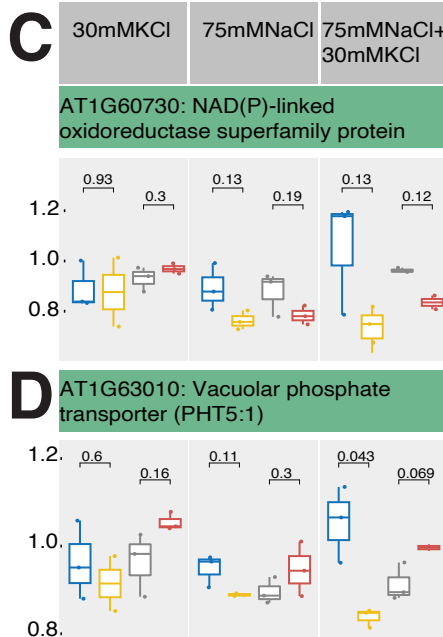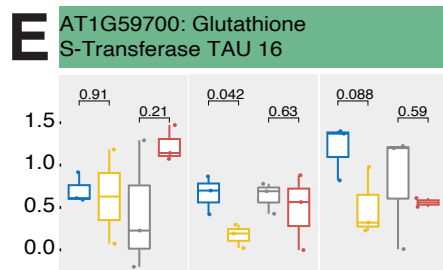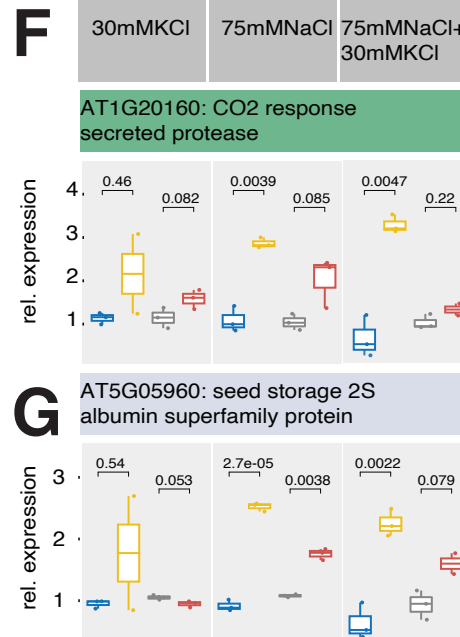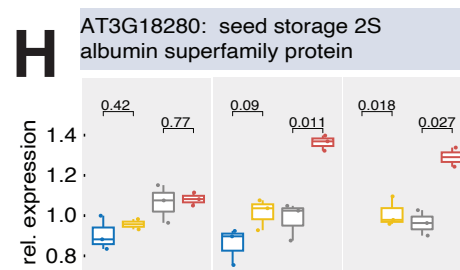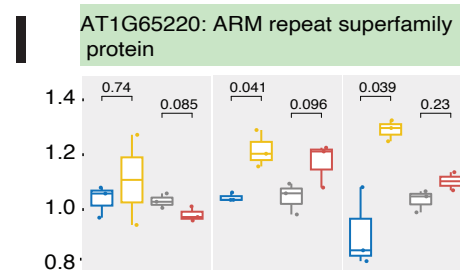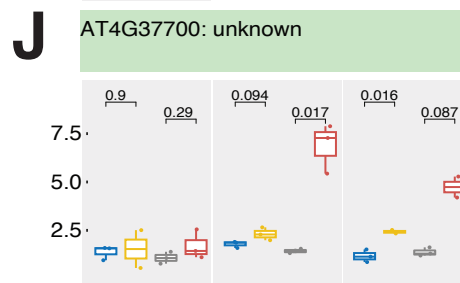

Supplement: S2 Fig — Additional expression profiles of genes identified to have conserved expression in response to salt and HKT1. The genes shared between the genetic backgrounds and treatments were inspected for their expression relative to control conditions across treatments, with (A-B) two transcripts to show significant reduction in response to both NaCl and NaCl + KCl treatment, (C-F) four transcripts to show significant reduction in response to NaCl + KCl, (G-H) two transcripts to show significant increase in response to both NaCl and NaCl + KCl treatments, and (I-J) two transcripts to show increase in response to NaCl + KCl treatment. The p-values listed above the individual comparisons between UAS-HKT1 and their background were calculated using T-test. (PDF) [file pgen.1011713.s002.pdf]

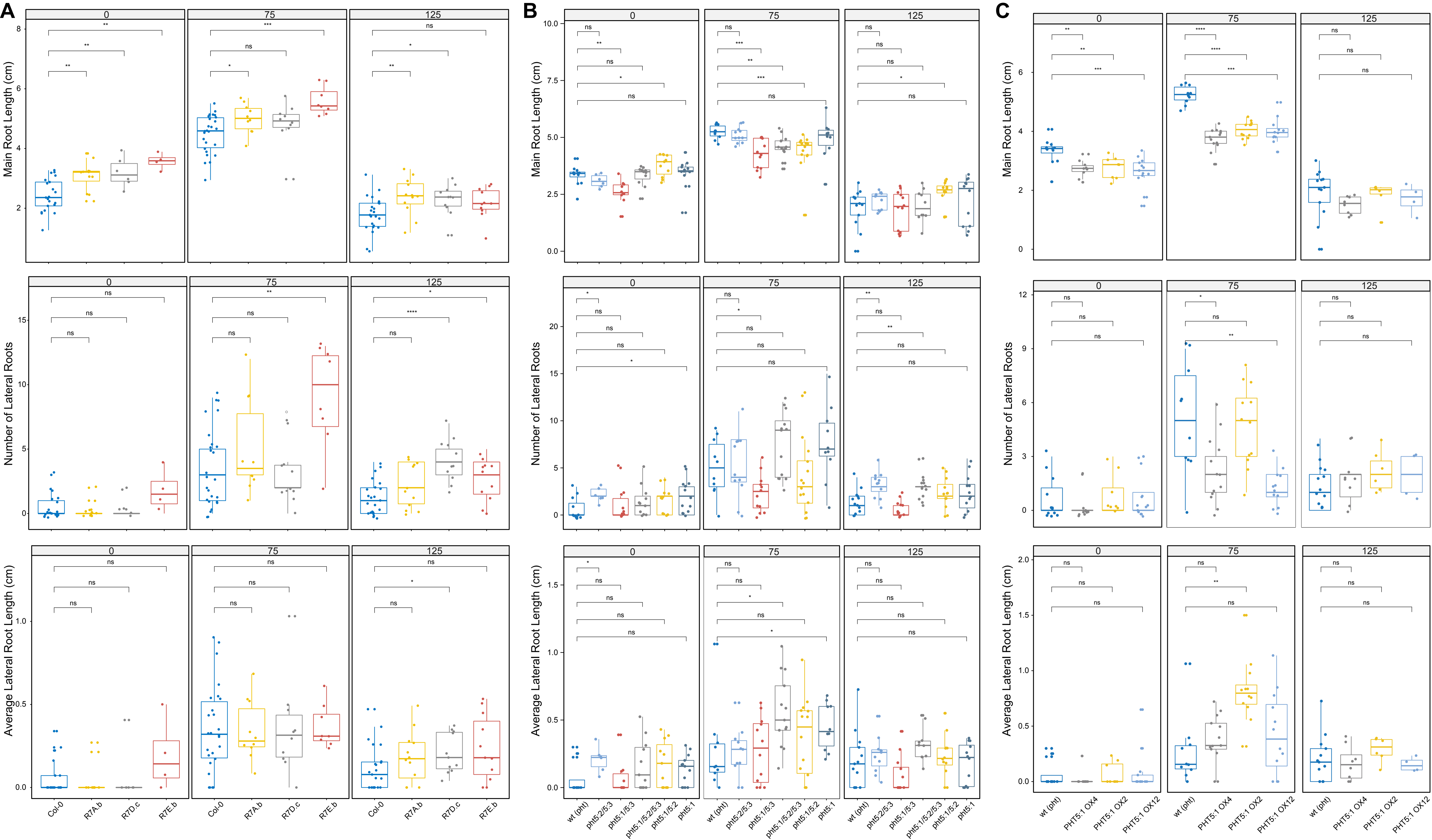

Supplement: S3 Fig — Root System architecture of (A) T-DNA insertion lines (SALK_023873, SAIL_789_E03 and SALK_098188C) (B) Single and multiple mutant lines obtained from Dr. Chiou Lab, and (C) gain-of-function liens obtained from Dr. Chiou Lab (T.-Y. Liu et al. 2016) [58]. The 4 days old seedlings were exposed to control (0 mM NaCl) or salt stress treatment (75 or 125 mM NaCl) for 4 (control) and 12 days (salt stress) of treatment prior to quantification of root architecture using SmartRoot. The graphs represent individual components of root architecture: Main Root Length, lateral root number and average lateral root length. The significant differences between individual mutant lines and their respective background lines were determined using one-way ANOVA test, with *, **, *** and **** indicating p-values below 0.05, 0.01, 0.001 and 0.0001 respectively. (PDF) [file pgen.1011713.s003.pdf]

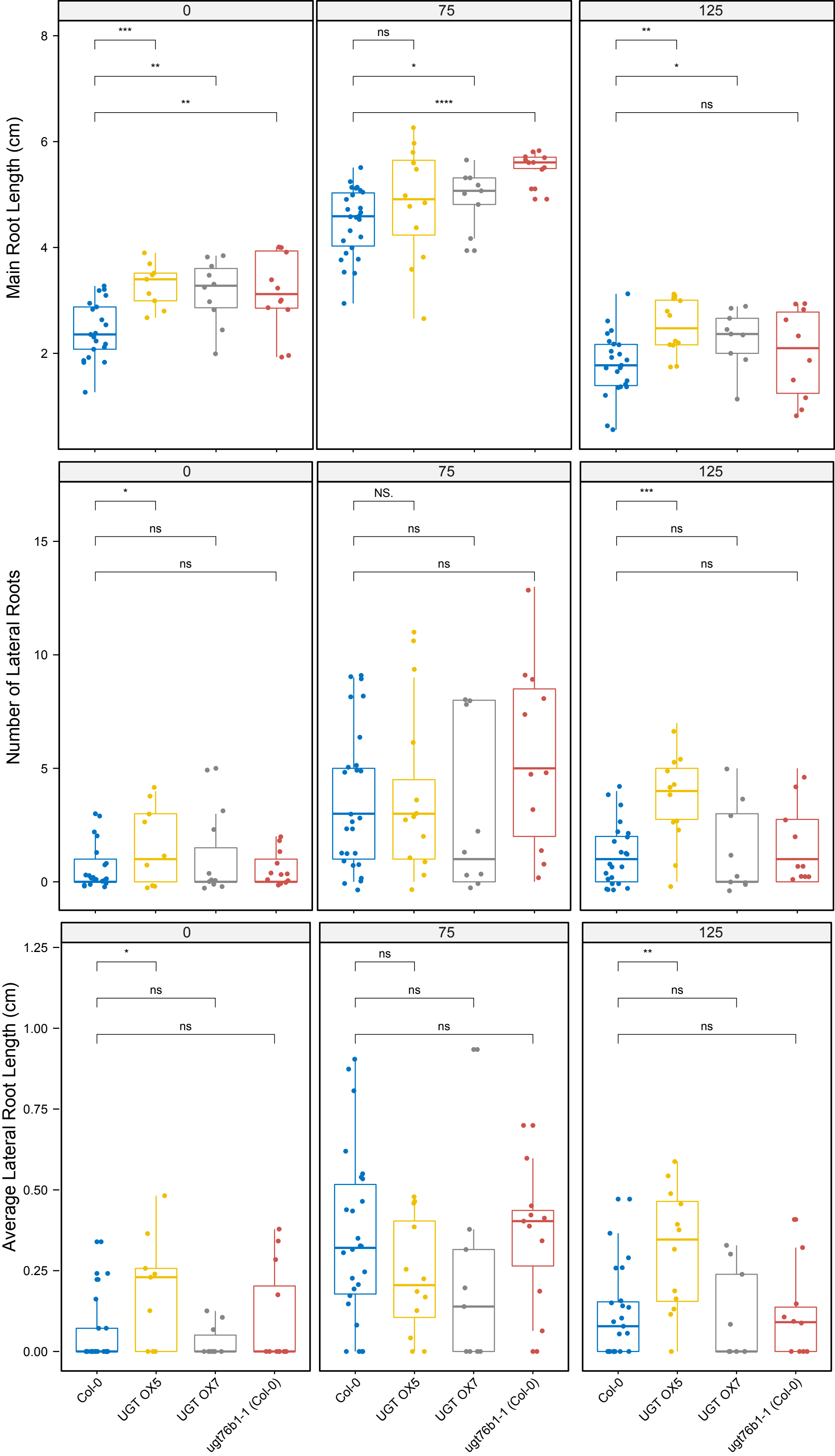

Supplement: S4 Fig — Root System architecture of two overexpression (OX) lines and one loss-of-function line obtained from Dr. Schaffner Lab [59]. The 4 days old seedlings were exposed to control (0 mM NaCl) or salt stress treatment (75 or 125 mM NaCl) for 4 (control) and 12 days (salt stress) of treatment prior to quantification of root architecture using SmartRoot. The graphs represent individual components of root architecture: Main Root Length, lateral root number and average lateral root length. The significant differences between individual mutant lines and their respective background lines were determined using one-way ANOVA test, with *, **, *** and **** indicating p-values below 0.05, 0.01, 0.001 and 0.0001 respectively. (PDF) [file pgen.1011713.s004.pdf]

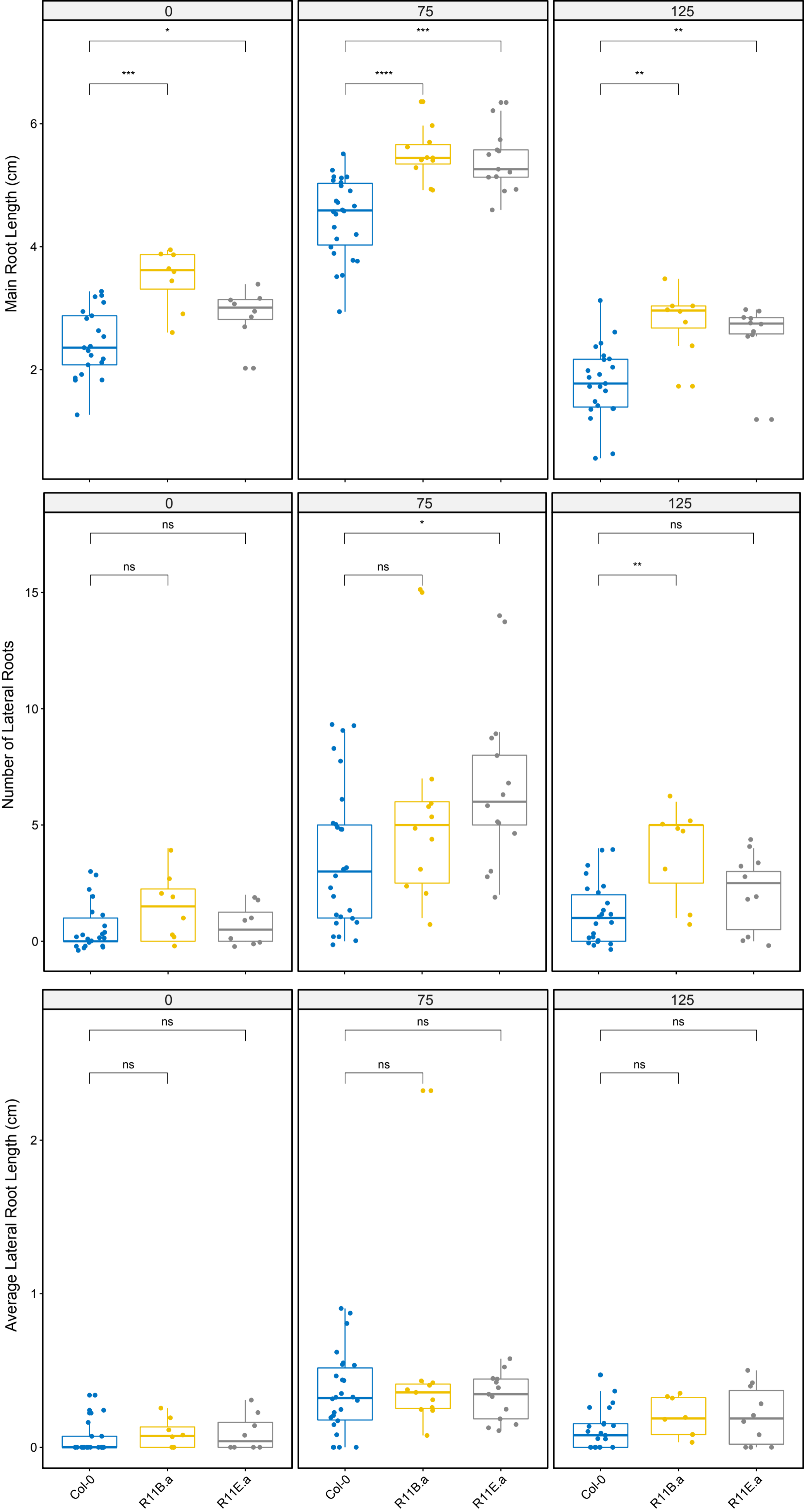

Supplement: S5 Fig — Root System architecture of T-DNA insertion lines (SALK_099861 and SALK_012112). The 4 days old seedlings were exposed to control (0 mM NaCl) or salt stress treatment (75 or 125 mM NaCl) for 4 (control) and 12 days (salt stress) of treatment prior to quantification of root architecture using SmartRoot. The graphs represent individual components of root architecture: Main Root Length, lateral root number and average lateral root length. The significant differences between individual mutant lines and their respective background lines were determined using one-way ANOVA test, with *, **, *** and **** indicating p-values below 0.05, 0.01, 0.001 and 0.0001 respectively. (PDF) [file pgen.1011713.s005.pdf]

**A**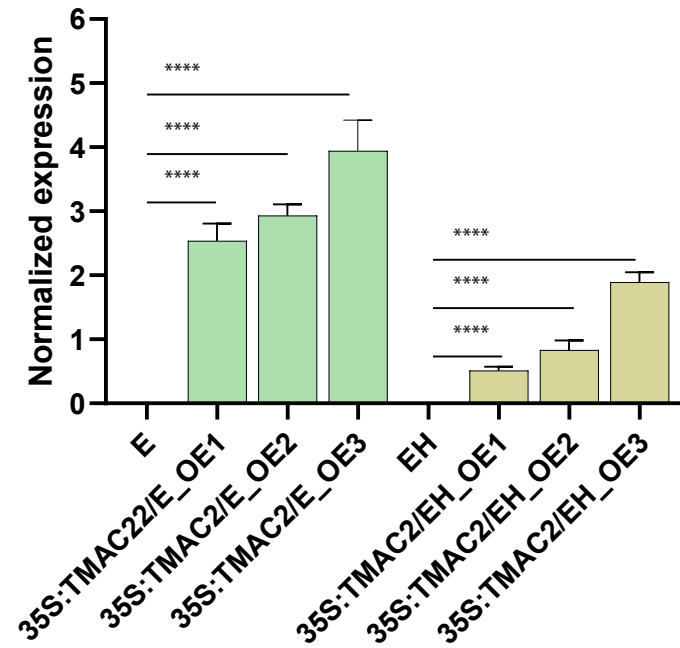**B**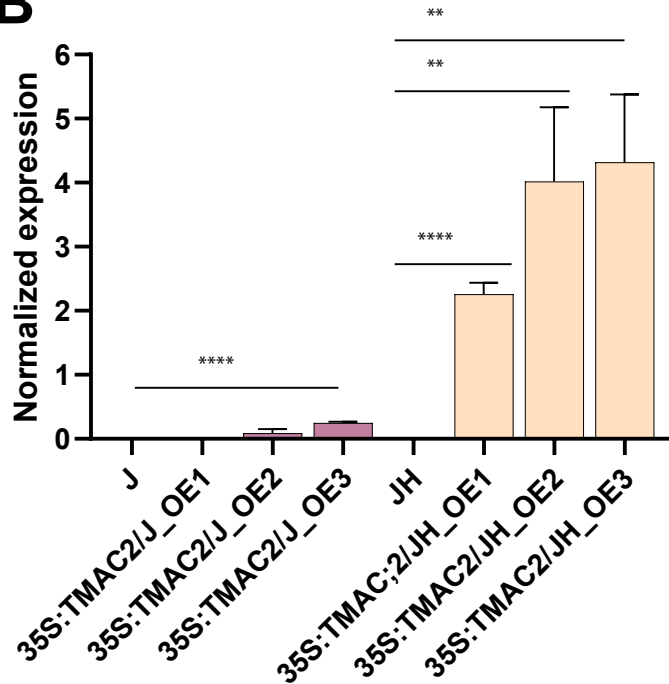

Supplement: S7 Fig — Expression of TMAC2 in Col-0 (E2586) and C24 (J2731) background with or without additional tissue-specific overexpression of HKT1. All expression results are based on three independent biological replicates collected from the leaves of soil grown plants. The significant differences between individual mutant lines and their respective background lines were determined using one-way ANOVA test, with *, **, *** and **** indicating p-values below 0.05, 0.01, 0.001 and 0.0001 respectively. (PDF) [file pgen.1011713.s007.pdf]

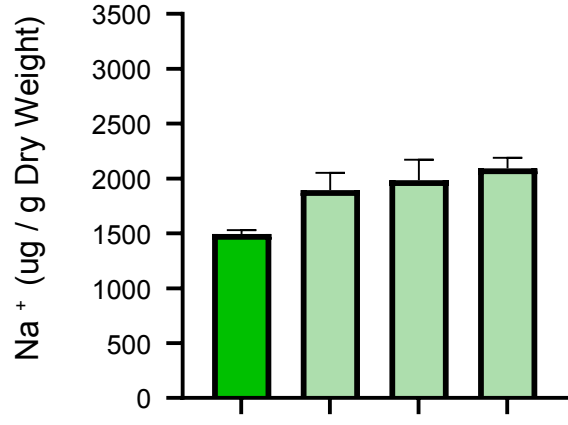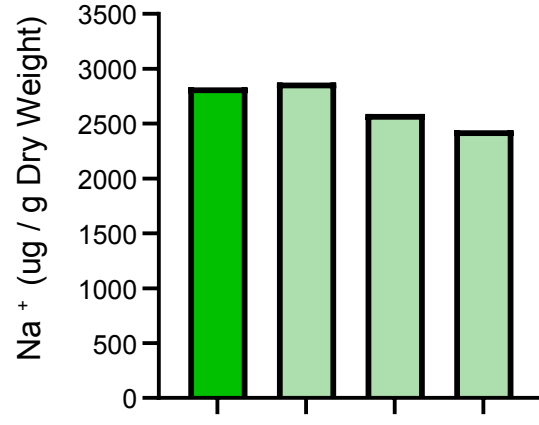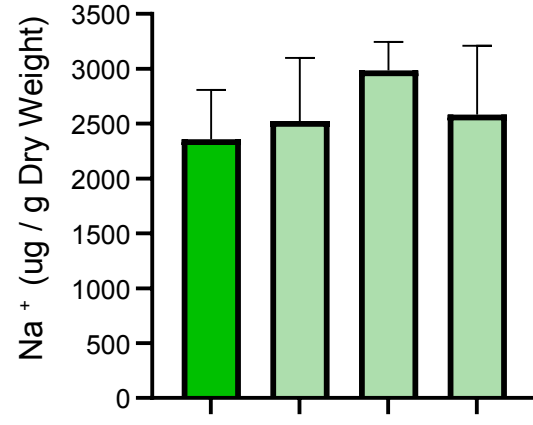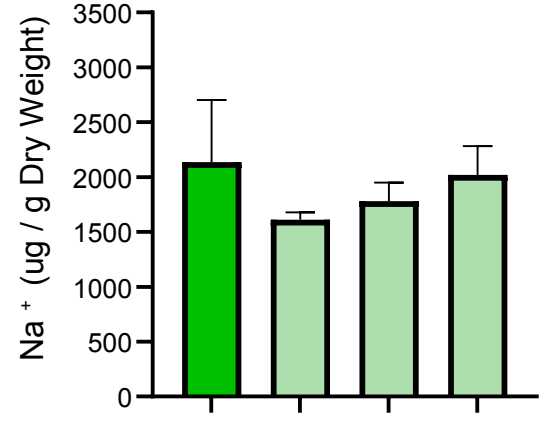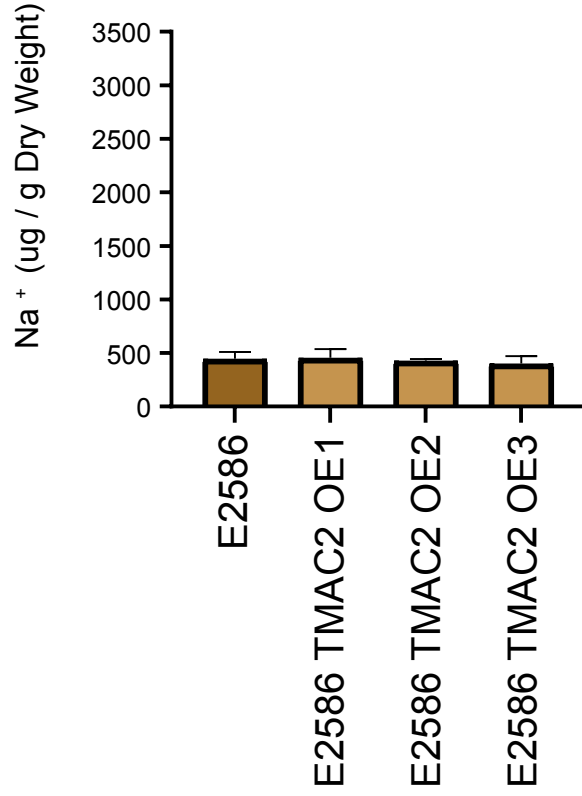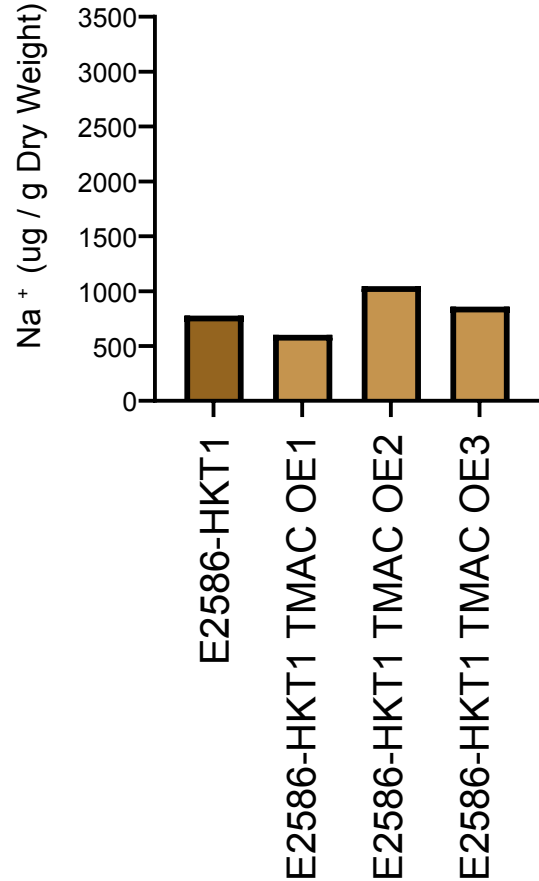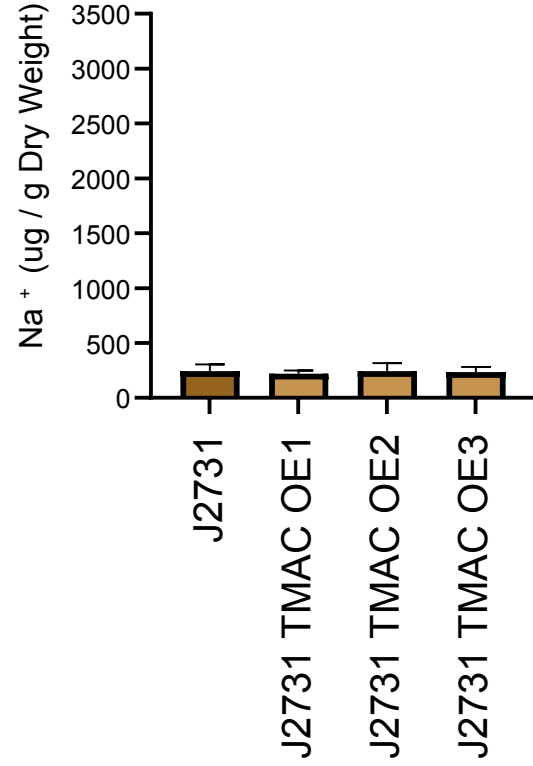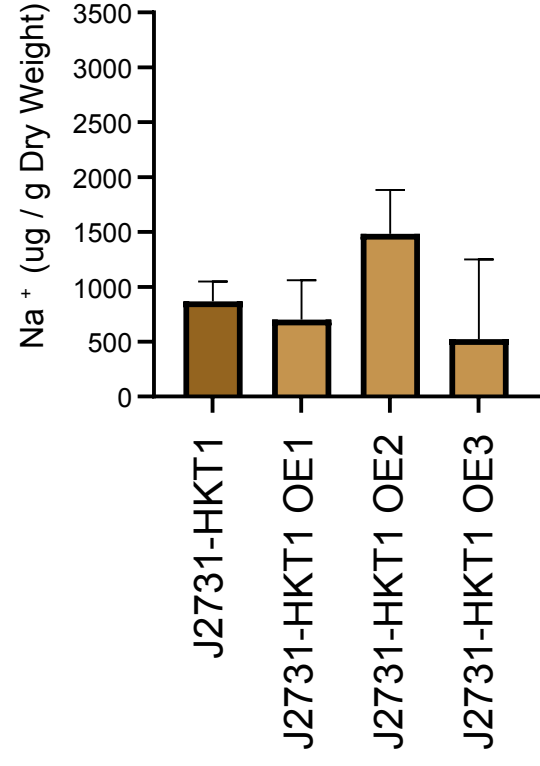

Supplement: S9 Fig — Sodium (Na+) accumulation in lines overexpressing TMAC2. Four days old Arabidopsis seedlings from lines with tissue-specific HKT1 overexpression in Col-0 (E2586) and C24 (J2731) backgrounds with and without TMAC2 overexpression were exposed to salt stress (75 mM NaCl) for 21 days. The sodium (Na+) accumulation was measured in seedling’s shoot (green graphs) and root tissue (brown graphs). The bars represent the mean value calculated from at least 20 seedlings, and the error bars represent standard error. The significant differences between individual mutant lines and their respective background lines were determined using one-way ANOVA test, with *, **, *** and **** indicating p-values below 0.05, 0.01, 0.001 and 0.0001 respectively. (PDF) [file pgen.1011713.s009.pdf]

**A**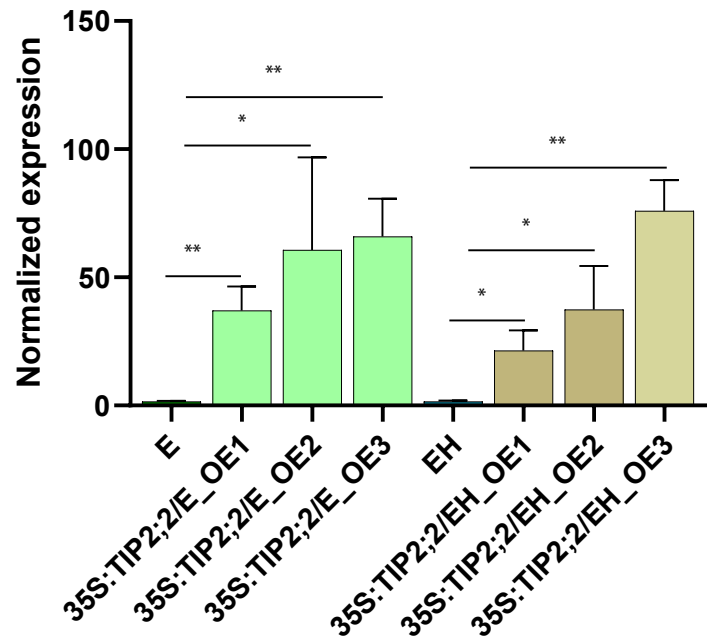**B**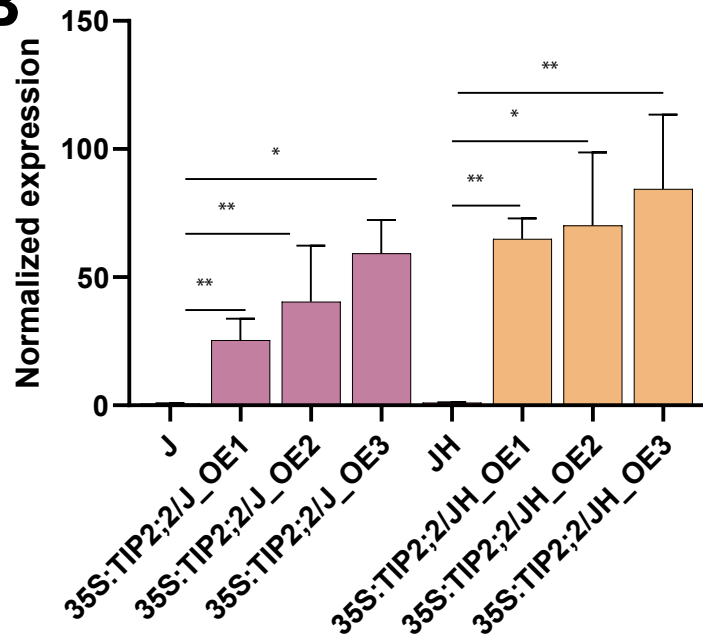**C**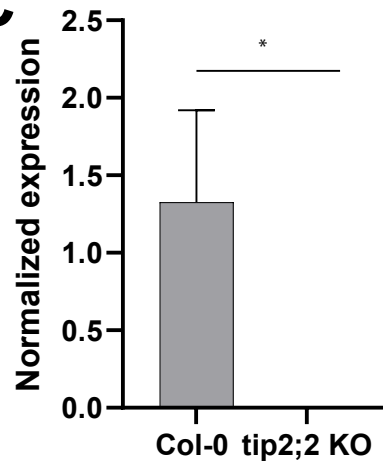

Supplement: S10 Fig — Expression of TIP2;2 in Col-0 (E2586) and C24 (J2731) background with or without additional tissue-specific overexpression of HKT1. All expressions are based on three independent biological replicates collected from the leaves of soil grown plants. The significant differences between individual mutant lines and their respective background lines were determined using one-way ANOVA test, with *, **, *** and **** indicating p-values below 0.05, 0.01, 0.001 and 0.0001 respectively. (PDF) [file pgen.1011713.s010.pdf]

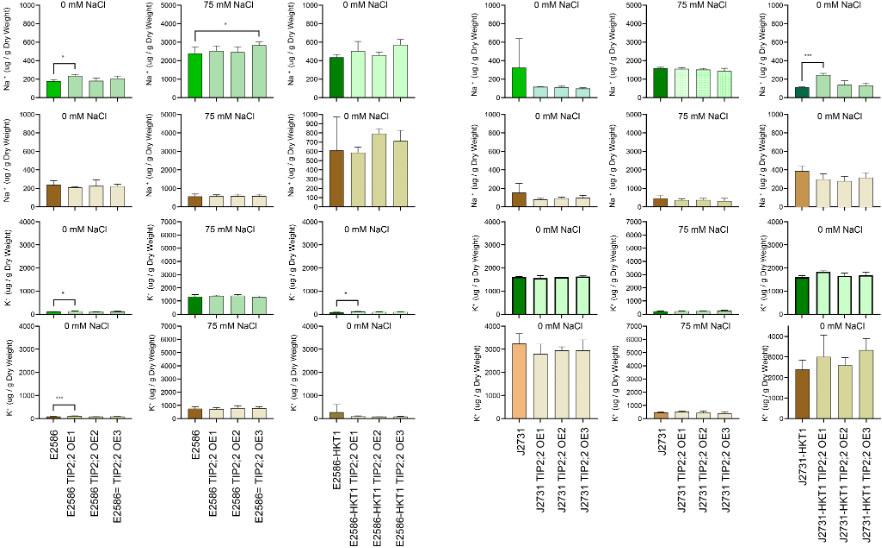

Supplement: S11 Fig — Sodium (Na+) and potassium (K+) accumulation under control (0 mM NaCl) and salt (75 mM NaCl) conditions of TIP2;2 overexpressing lines. Four days old Arabidopsis seedlings from lines with tissue-specific HKT1 overexpression in Col-0 (E2586) and C24 (J2731) backgrounds with and without TIP2;2 overexpression were exposed to salt stress (75 mM NaCl) for 21 days. The sodium (Na+) and potassium (K+) accumulation was measured in seedling’s shoot (green graphs) and root tissue (brown graphs). The bars represent the mean value calculated from at least 20 seedlings, and the error bars represent standard error. The significant differences between individual mutant lines and their respective background lines were determined using one-way ANOVA test, with *, **, *** and **** indicating p-values below 0.05, 0.01, 0.001 and 0.0001 respectively. (PNG) [file pgen.1011713.s011.png]

**A**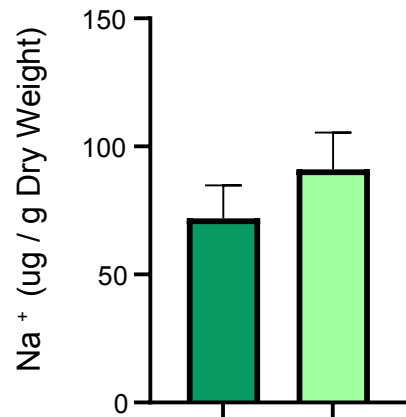**B**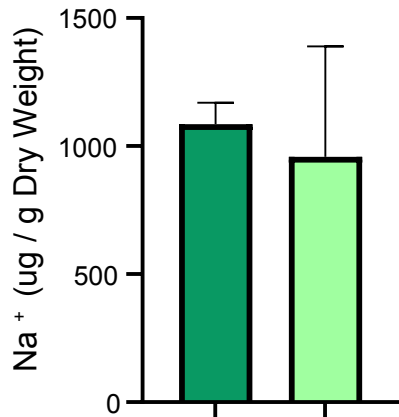**C**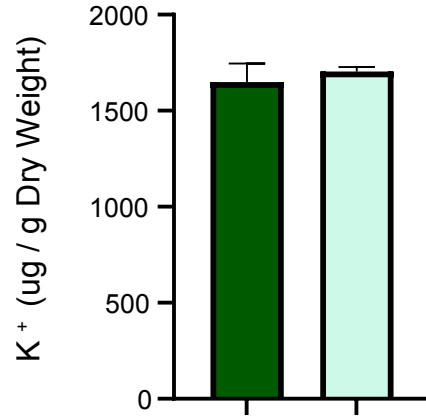**D**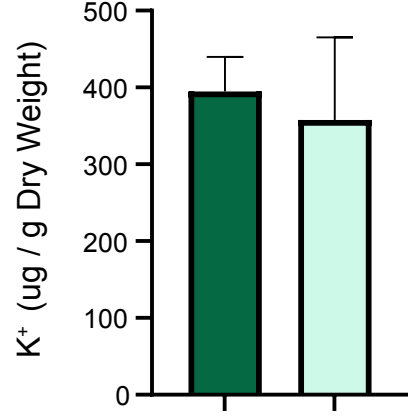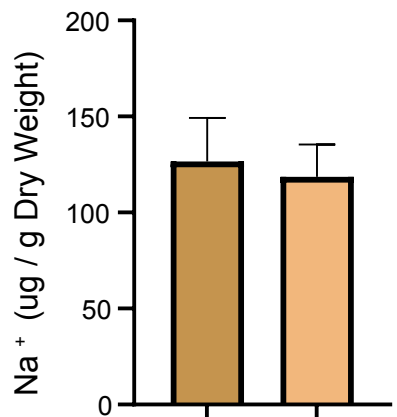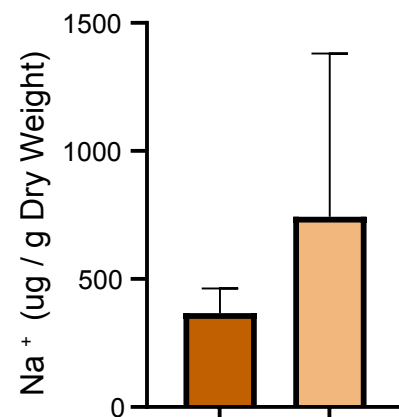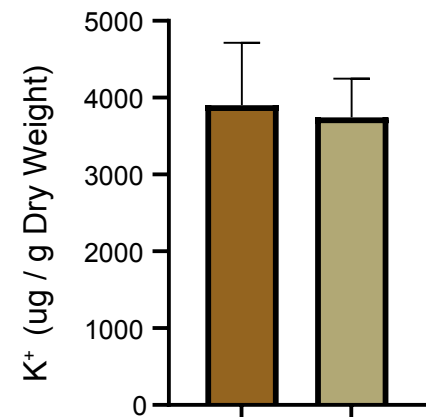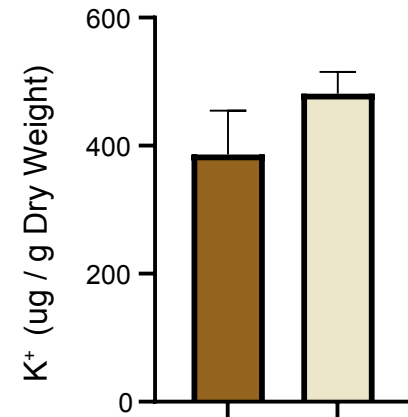Col-0 *tip2;2*Col-0 *tip2;2*Col-0 *tip2;2*Col-0 *tip2;2*

Supplement: S12 Fig — Sodium (Na+) and potassium (K+) accumulation under control and salt stress conditions in tip2;2 mutant lines. Four days old Arabidopsis seedlings from Col-0 and tip2;2 T-DNA insertion line (SALK_152463) were exposed to control (0 mM NaCl) or salt stress (75 mM NaCl) for 21 days. The sodium (Na+) accumulation was measured in seedling’s shoot (green graphs) and root tissue (brown graphs) under (A) control or (B) salt stress conditions. Additionally, potassium (K+) accumulation was also measured in seedling’s shoot (green graphs) and root tissue (brown graphs) under (C) control or (D) salt stress conditions. The bars represent the mean value calculated from at least 20 seedlings, and the error bars represent standard error. The significant differences between individual mutant lines and their respective background lines were determined using one-way ANOVA test, with *, **, *** and **** indicating p-values below 0.05, 0.01, 0.001 and 0.0001 respectively. (PDF) [file pgen.1011713.s012.pdf]

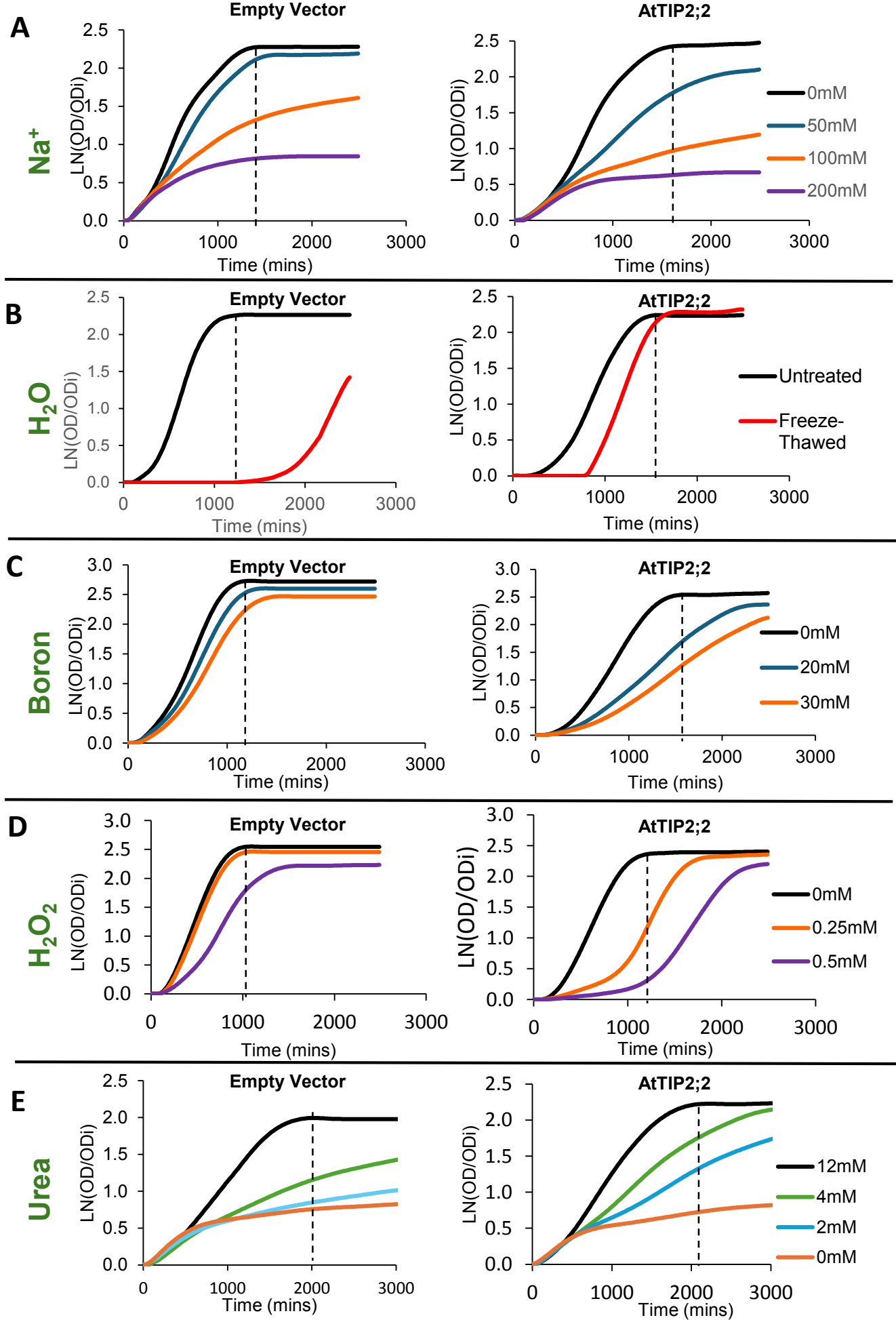

Supplement: S13 Fig — Respective yeast cultures were exposed to (A) 0mM, 50mM, 100MM and 200mM NaCl, for sodium toxicity assay; (B) Untreated and Freeze-thawed treated for Water transport assay; (C) 0mM, 20mM and 30mM Boric acid for Boron toxicity assay; (D) 0mM, 0.25mM and 0.5mM H2O2, for H2O2 toxicity assay and (E) 12mM, 4mM, 2mM and 0mM Urea for urea growth-based screen. Growth comparisons shown in Fig 7 were captured from the area under the curves (AUC) until the vertical dashed lines (measuring time point) show on Ln(OD/ODi) vs. time graphs. AtTIP2;2- expressing yeast displays reduced growth over time at each of the sodium, boric acid, H2O2 concentrations, indicative of an increased sensitivity/toxicity response to these treatments compared to Empty vector control. AtTIP2;2-expressing yeast also shows increased survivorship/recovery post freeze-thaw treatment, compared to Empty vector control which shows minimal growth post treatment exposure. AtTIP2;2-expressing yeast showed enhanced yeast growth over time at low nitrogen media concentrations (2mM and 4mM urea) compared to Empty vector control. (PDF) [file pgen.1011713.s013.pdf]
