# Supplementary material for: Root remodeling mechanisms and salt tolerance trade-offs: The roles of HKT1, TMAC2, and TIP2;2 in Arabidopsis
Source: PLoS Genet. 2025 Jun 11;21(6):e1011713. doi: 10.1371/journal.pgen.1011713 (PMC12204623; doi:10.1371/journal.pgen.1011713)
Supplement: S8 Fig — The ABA accumulation in Col-0 (E2586) and C24 (J2731) seedlings with and without tissue specific overexpression of HKT1 and additional over-expression of TMAC2 was measured in shoots (green graphs) and roots (brown graphs) Arabidopsis seedlings 21 days after transfer to 0 or 75 mM NaCl. (PDF) [file pgen.1011713.s008.pdf]

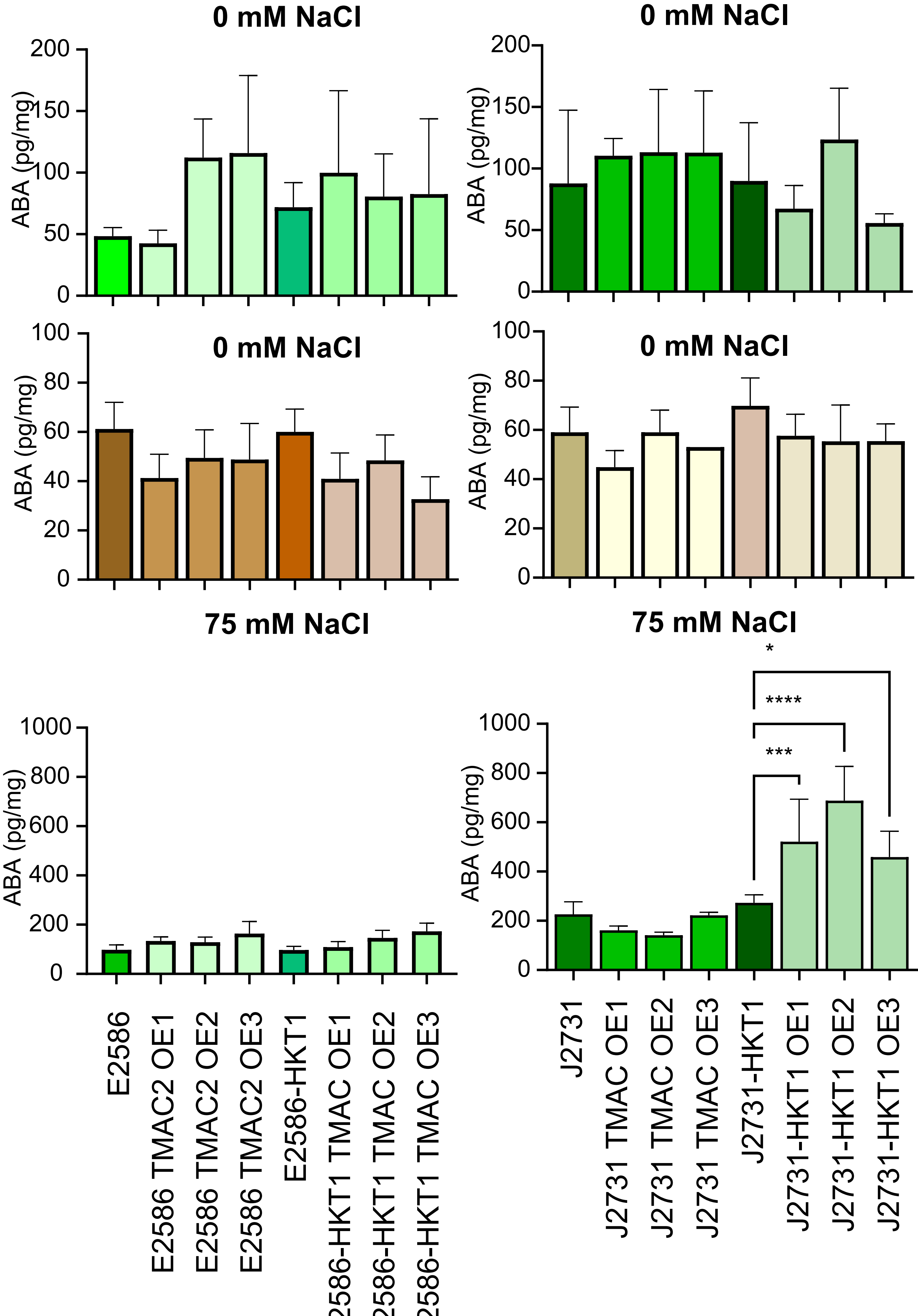

0 mM NaCl
ABA (pg/mg)
0 mM NaCl
ABA (pg/mg)
0 mM NaCl
ABA (pg/mg)
0 mM NaCl
ABA (pg/mg)
75 mM NaCl
ABA (pg/mg)
75 mM NaCl
ABA (pg/mg)
*
****
***
E2586
E2586 TMAC2 OE1
E2586 TMAC2 OE2
E2586 TMAC2 OE3
E2586-HKT1
2586-HKT1 TMAC OE1
2586-HKT1 TMAC OE2
2586-HKT1 TMAC OE3
J2731
J2731 TMAC OE1
J2731 TMAC OE2
J2731 TMAC OE3
J2731-HKT1
J2731-HKT1 OE1
J2731-HKT1 OE2
J2731-HKT1 OE3
