# Supplementary material for: Root remodeling mechanisms and salt tolerance trade-offs: The roles of HKT1, TMAC2, and TIP2;2 in Arabidopsis
Source: PLoS Genet. 2025 Jun 11;21(6):e1011713. doi: 10.1371/journal.pgen.1011713 (PMC12204623; doi:10.1371/journal.pgen.1011713)
Supplement: Attachment — Submitted filename: TMAC2 review.pdf [file pgen.1011713.s020.pdf]

This manuscript describes some interesting new results regarding the role of TMAC2 (a negative ABA-regulator) and of a tonoplast aquaporin-type transporter (TIP2.2) in lateral root inhibition under salt stress. The data are interesting and new. However, I struggled with some of the text especially in the Introduction and the early part of the Results. I did not completely grasp the precise question addressed (role of HKT1?) and certainly lost sight of it early on. My comments below just concentrate on these aspects, but the whole paper should be edited to follow a clear line of hypothesis testing.

The Introduction needs a better description of the expression patterns of the overexpressed HKT1 in J2731 and E2586 and of the corresponding phenotypes. If I understand correctly from the previous publications both lines overexpress HKT1 in the stele but overexpression includes/excludes the pericycle in J2731 and E2586. (This is repeated in this manuscript but legend of Fig. 6 in Julkowska et al (Plant Cell, 2017) notes "UAS-HKT1 lines, with enhanced *HKT1* expression in the root pericycle", I assume this is a mistake?).

Moller et al reported a decrease in shoot Na for both overexpression lines but the effect is stronger in J2731; UASGAL4:HKT1 than in E2586; UASGAL4:HKT (mentioned in the Introduction of this manuscript). The Plant Cell Julkowska et al 2017 paper (Fig. 6) showed inhibition of LR development/growth in both overexpression lines but this phenotype is stronger in E2586; UASGAL4:HKT than in J2731; UASGAL4:HKT1 (not mentioned in the Instroductio of this manuscript). Is this correct?

If so, could the authors please justify the hypothesis that the effect on lateral root development is due to Na accumulation in the pericycle. Would the latter not be expected to be stronger in J2731; UASGAL4:HKT1 than in E2586; UASGAL4:HKT.?

The precise research question (how does HKT1 alter LR development?) should be explicit, and then followed through in the Results and Discussion.

Results:

I find the initial description of the transcriptome data distracts from the research question. For example, enrichment of annotations for some pairwise comparisons shown in Fig 1 seems to be only of tangential interest for this paper. Given the multi-factorial design of the experiment a more sophisticated analysis of the dataset would be possible and could be published separately. Here, Fig. 1B-C and their description could be omitted or moved to Supplemental material. I also find some of the conclusions unjustified. E.g. that a stronger transcriptional response in the Na+K treated plants compared to Na alone supports the observation that addition of K alleviates that root phenotype. One could just as well expect a weaker response given that the plants are less stressed.

From line 335 onwards there is more focus on the research question. To ‘identify the genes downstream of UAS-HKT1 that may be involved in the repression of lateral root development under salt stress’ the authors extract genes that show interesting differences in the response profiles between the lines. This makes sense. However, I do not understand the term ’similar’ in the legend of Fig. 2 (clearly the response patterns differ). More importantly, I find it difficult to match the profiles with the reported root phenotypes (see my comment on Introduction). Naiively, I would have looked for genes that show a similar difference to WT in both over-expression lines (as they are both inhibited in LR development) but with a stronger difference n E2586 than in J2731 background (see Fig. 6 in Julkowska et al 2017)) . Some genes show such pattern, others show a stronger response in J2731 (matching the Na removal phenotype reported in Moller) while TMAC actually has an opposite profile in J2731 and E2586. Why did the authors focus on this gene? It would be good to sort genes into different profiles and then systematically compare them with the reported phenotypes.

The Discussion could be better focussed. Hypothetical statements, especially not linked to the genes investigated should be omitted.

I struggle to understand what exactly is being concluded. Yes, clearly gene functions and responses differ in the two genetic backgrounds, which is probably not too surprising but what does this mean? Could it be that the different root phenotypes in the HKT1 overexpression lines are not due to differences in HKT1 localisation but to other differences in the two backgrounds?

The authors try to explain their observations with ‘feedback loops’ and ‘complex regulatory networks’. A scheme depicting at least a working hypothesis would certainly help the reader.
