# Supplementary material for: Root remodeling mechanisms and salt tolerance trade-offs: The roles of HKT1, TMAC2, and TIP2;2 in Arabidopsis
Source: PLoS Genet. 2025 Jun 11;21(6):e1011713. doi: 10.1371/journal.pgen.1011713 (PMC12204623; doi:10.1371/journal.pgen.1011713)
Supplement: Attachment — Submitted filename: Response to Reviewers.docx [file pgen.1011713.s022.docx]

*Reviewer #1 comment: In this work, Alshareef et al investigated that role of TMAC2 (a negative transcriptional regulator of ABA) in lateral root development in two Arabidopsis ecotypes. The authors reported that overexpression of TMAC2 modulated root ABA content, affecting lateral root development, and that these changes were ecotype-specific. It is concluded that HKT1 and TMAC2 may form a positive feedback loop in ABA signalling operating upstream of lateral root development.
I must confess I have rather mixed feelings about this work. On one hand, the paper addresses a very important topic – plant adaptation to salinity stress, and the studies on the role of root system architecture in this process are relatively few and investigated much less that for other adaptive traits, such as Na exclusion or stomata patterning. On the other hand, the paper reads like a “dog’s breakfast” and mixes together various components without proving the existence of any casual relationship between them.

My first major issue is with an attempt to make HKT1 operation central to this story. With a due respect, this transporter is only one (of many) that determine tissue Na content. Thus, the entire working hypothesis per se looks rather vague to me.*

Response: We thank the reviewer for their thoughtful evaluation of our work and constructive comments. We appreciate the reviewer’s comments about the manuscript's structure and have tried to address them to the best of our abilities.

We acknowledge the concern that HKT1 should not be framed as the sole determinant of Na⁺ homeostasis in plant tissues. However, our rationale for focusing on HKT1 in the context of lateral root development is based on the distinct effect it has on root system architecture—an aspect not commonly observed with other ion transporters. Unlike SOS1, GORK, or other known salt-responsive transporters, which primarily influence main root length, HKT1 was identified in screens for natural variation in root architecture (Julkowska et al., 2017), suggesting a previously unrecognized role in modulating lateral root development under salt stress.

Importantly, HKT1 mutants do not exhibit a strong lateral root phenotype under non-stress conditions, but tissue-specific HKT1 gain-of-function expression led to a severe inhibition of lateral root development under salt stress. This suggests that HKT1’s effect on root system architecture is conditional and linked to specific stress responses. We have revised the manuscript to more clearly articulate this rationale in the introduction and throughout the results, ensuring that our working hypothesis is better defined and less reliant on assumptions about HKT1’s centrality.

Finally, we recognize the reviewer’s concerns regarding the manuscript structure and the need for a clearer causal narrative. We have made significant efforts to streamline the manuscript, improve clarity, and explicitly justify the logical connections between components, ensuring a more cohesive presentation of our findings.

*Reviewer #1 comment: The authors then add two different ecotypes to the story, and report rather opposite effects of TMAC2 on lateral root development. While overexpression of TMAC2 in the Col-0 background increased ABA accumulation, its overexpression in C24 reduced ABA accumulation. Calling this a “genotype-specific effect” does not explain much and merely a way of confirming that the authors could not explain the reasons between these controversial results. The same is also true for the causal link between ABA and lateral root development. Many other factors (e.g., ethylene, ROS, etc) maya also contribute to this process. So, the entire story is outstretched, and conclusions are not substantiated by the data.*

Response: We appreciate the reviewer’s concerns and the opportunity to clarify our approach. Our inclusion of two ecotypes was not an attempt to introduce unnecessary complexity but rather a deliberate strategy to examine the conservation and variability of root system responses to salt stress across genetic backgrounds. Both Col-0 and C24 exhibit reduced lateral root development under salt stress, and identifying overlapping transcriptional responses was central to our study.

The observed differences in TMAC2’s effect on ABA accumulation between Col-0 and C24 are not contradictions but rather insights into genotype-specific regulatory networks. Instead of dismissing this as noise, we view it as evidence that different genetic backgrounds may engage distinct molecular mechanisms to achieve similar phenotypic outcomes. We have revised the manuscript to ensure that this rationale is explicitly stated and that the logic behind our comparisons is clearer.

We fully acknowledge that ABA is not the sole regulator of lateral root development under stress, and we do not discount the roles of other hormones such as ethylene, ROS, and auxin. However, our transcriptomic and functional analyses identified ABA-related genes as key players in the observed phenotype, making them a central focus of this study. We have refined our discussion to acknowledge additional potential regulatory factors while ensuring that our conclusions remain grounded in the data.

*Reviewer #1 comment: In the light of this, I believe the authors should be less bias in their assumption of HKT1 being central to lateral root development and provide more comprehensive and less subjective approach to investigate the causal link between ABA signalling and RSA formation. This implies omitting some (not directly relevant) data and conducting additional experimentation.*

*Response:* We appreciate the reviewer’s thoughtful feedback. Our findings demonstrate that HKT1’s effect on lateral root development is conserved across two distinct genetic backgrounds, reinforcing its role in root system architecture under salt stress. However, the mechanisms linking HKT1 to lateral root development appear genotype-specific, highlighting the complexity of salt stress tolerance and its interactions with other physiological traits. This variability is not a limitation but rather an important insight into the genetic regulation of stress responses.

Regarding the suggestion to omit “not directly relevant” data, we firmly believe that removing these findings would weaken the broader perspective we aim to provide—that both conserved and genotype-dependent mechanisms influence salt stress adaptation. The observed differences between Col-0 and C24 offer a valuable case study on natural variation in stress responses, which is critical for understanding the complexity of salt tolerance strategies. Nevertheless, we addressed this concern and aimed to streamline the manuscript move by moving more of the not-directly relevant results into supplemental figures.

As for additional experimentation, while we recognize the value of further validation, such work is beyond the current scope of this manuscript. Many co-authors have since transitioned to new roles, and the proposed experiments would require substantial time, resources, and funding that are not presently available. Nonetheless, we hope our study lays the foundation for future investigations into the intricate links between ion homeostasis, ABA signaling, and root system architecture under salinity stress.

*Minor issues:
- Ln 28. Describe TMAC2 abbreviation.*

We have now included full description of TMAC2 abbreviation

*- Ln 50-55. This is a rather eclectic list, mixing together various traits and processes, without any structural logic.*

We have now re-written this fragment of the Introduction, with more logical order and explanations behind the listed traits and processes.

*- Ln 79 and elsewhere. “Johannes D. Scharwies et al 2024”. Why using first names in references?! This looks like a student’s style of writing to me. Have any of co-authors read and approved the MS? …*

Our citation formatting software made this mistake. That specific citation refers to Johannes Scharwies’s thesis, and probably the type of reference was causal to mis-citation. Thank you for spotting this. We have now carefully checked all of the citations that they have uniform format.

Also - yes - all co-authors have read and approved the manuscript.

*Reviewer #2 comment: This manuscript describes some interesting new results regarding the role of TMAC2 (a negative ABA-regulator) and of a tonoplast aquaporin-type transporter (TIP2.2) in lateral root inhibition under salt stress. The data are interesting and new. However, I struggled with some of the text especially in the Introduction and the early part of the Results. I did not completely grasp the precise question addressed (role of HKT1?) and certainly lost sight of it early on. My comments below just concentrate on these aspects, but the whole paper should be edited to follow a clear line of hypothesis testing.*

*Response:* We thank the reviewer for their thoughtful and constructive comments. We have not sufficiently articulated our hypothesis in the initial version of the manuscript. We have worked throughout the manuscript to explicitly explain the *raison d’etre* for this manuscript, including stating the hypothesis and reasons for individual comparisons.

*Reviewer #2 comment: The Introduction needs a better description of the expression patterns of the overexpressed HKT1 in J2731 and E2586 and of the corresponding phenotypes. If I understand correctly from the previous publications both lines overexpress HKT1 in the stele but overexpression includes/excludes the pericycle in J2731 and E2586. (This is repeated in this manuscript but legend of Fig. 6 in Julkowska et al (Plant Cell, 2017) notes “UAS-HKT1 lines, with enhanced HKT1 expression in the root pericycle”, I assume this is a mistake?).*

*Response:* It is indeed correct that the difference between J2731 and E2586 was not discussed in much detail in Julkowska et al., 2017. We have now stressed that the differences in overexpression patterns between the lines might be an additional factor contributing to transcriptomics changes.

*Reviewer #2 comment: Moller et al reported a decrease in shoot Na for both overexpression lines but the effect is stronger in J2731; UASGAL4:HKT1 than in E2586; UASGAL4:HKT (mentioned in the Introduction of this manuscript). The Plant Cell Julkowska et al 2017 paper (Fig. 6) showed inhibition of LR development/growth in both overexpression lines but this phenotype is stronger in E2586; UASGAL4:HKT than in J2731; UASGAL4:HKT1 (not mentioned in the Instroductio of this manuscript). Is this correct?*

*Response:* We appreciate the reviewer’s close reading of the literature and their request for clarification regarding the effects of HKT1 overexpression on shoot Na⁺ accumulation and root system architecture in the J2731 (C24) and E2586 (Col-0) lines.

It is correct that HKT1 overexpression led to a stronger reduction in shoot Na⁺ content in J2731 (C24) compared to E2586 (Col-0), as reported by Møller et al., 2009. However, regarding lateral root development, our previous work (Julkowska et al., 2017) found that the inhibition of lateral root growth was of the same magnitude in both backgrounds, even though the main root length was more severely affected in Col-0 (E2586) than in C24 (J2731).

The difference in main root length reduction in E2586 vs. J2731 could be due to either genetic background differences or the specific HKT1 overexpression pattern in these lines. However, as C24 naturally has a shorter root system, it is challenging to disentangle whether the observed differences stem from intrinsic genetic variation or HKT1-mediated effects.

Given that HKT1 overexpression had a consistent effect on lateral root inhibition across both backgrounds, we focused our study on this specific phenotype, which was the most robust across conditions. For this reason, we omitted further discussion of the main root length differences in the manuscript to maintain clarity and focus on the most relevant findings. However, we have now revised the Introduction to explicitly mention this distinction, ensuring that the context is clear for the reader.

*Reviewer #2 comment: If so, could the authors please justify the hypothesis that the effect on lateral root development is due to Na accumulation in the pericycle. Would the latter not be expected to be stronger in J2731; UASGAL4:HKT1 than in E2586; UASGAL4:HKT.?*

*Response:* We appreciate the reviewer’s insightful question and the opportunity to clarify our hypothesis. We have now explicitly stated our hypothesis and revised the manuscript accordingly to ensure clarity.

Regarding the reviewer’s suggestion, while one might expect a stronger effect on lateral root development in J2731 (C24) due to pericycle-specific HKT1 expression, our data indicate that the inhibition of lateral root development is of similar magnitude in both J2731 and E2586. This suggests that Na⁺ accumulation in the pericycle alone may not fully explain the observed phenotype, and additional factors, such as ABA signaling or other tissue-specific regulatory mechanisms, may also play a role.

To address this, we have refined the manuscript to ensure that our reasoning and conclusions are clearly articulated, making the logical basis of our hypothesis more explicit throughout.

*Reviewer #2 comment: The precise research question (how does HKT1 alter LR development?) should be explicit, and then followed through in the Results and Discussion.*

*Response:* This is a great suggestion. Thank you for this. We have now explicitly stated the hypothesis explored in this manuscript throughout the manuscript *Reviewer #2 comment: Results: I find the initial description of the transcriptome data distracts from the research question. For example, enrichment of annotations for some pairwise comparisons shown in Fig 1 seems to be only of tangential interest for this paper. Given the multifactorial design of the experiment a more sophisticated analysis of the dataset would be possible and could be published separately. Here, Fig. 1B-C and their description could be omitted or moved to Supplemental material. I also find some of the conclusions unjustified. E.g. that a stronger transcriptional response in the Na+K treated plants compared to Na alone supports the observation that addition of K alleviates that root phenotype. One could just as well expect a weaker response given that the plants are less stressed.*

*Response:* We appreciate the reviewer’s feedback and agree that the initial description of the transcriptome data can be streamlined to maintain focus on the research question. To address this, we have moved parts of Fig. 1 to the supplemental figures and substantially shortened the discussion on overall transcriptional patterns.

Additionally, we have removed the conclusion suggesting that a stronger transcriptional response in Na⁺+K⁺ treated plants directly supports the alleviation of the root phenotype, as we acknowledge that alternative interpretations are possible. These revisions ensure that the manuscript remains concise and directly relevant to the core findings.

*Reviewer #2 comment: From line 335 onwards there is more focus on the research question. To ‘identify the genes downstream of UAS-HKT1 that may be involved in the repression of lateral root development under salt stress’ the authors extract genes that show interesting differences in the response profiles between the lines. This makes sense. However, I do not understand the term ’similar’ in the legend of Fig. 2 (clearly the response patterns differ). More importantly, I find it difficult to match the profiles with the reported root phenotypes (see my comment on Introduction).*

*Response:* We appreciate the reviewer’s careful assessment and the opportunity to clarify our approach. Our use of the term "similar" was intended to convey the identification of conserved transcriptional responses across both backgrounds (Col-0 and C24) rather than implying identical expression patterns. The goal was to highlight genes that exhibit a consistent regulatory trend in response to salt stress and HKT1 overexpression, despite potential differences in magnitude or directionality between backgrounds.

To improve clarity, we have revised the manuscript to better explain how we generated gene lists and made our comparisons. Specifically, we have now explicitly stated that our analysis aimed to identify genes that showed conserved responses to salt stress and tissue-specific HKT1 overexpression in both backgrounds, which may underlie shared mechanisms regulating lateral root development.

Additionally, we acknowledge that the current figure legend could be misleading. To better reflect the intent of this analysis, we have consolidated Fig. 1 and Fig. 2 and provided it with alternative figure legend title.

This revision should more clearly convey the focus on identifying shared transcriptional responses across genetic backgrounds, while maintaining consistency with the overall research question.

*Reviewer #2 comment: Naiively, I would have looked for genes that show a similar difference to WT in both over-expression lines (as they are both inhibited in LR development) but with a stronger difference n E2586 than in J2731 background (see Fig. 6 in Julkowska et al 2017)) . Some genes show such pattern, others show a stronger response in J2731 (matching the Na removal phenotype reported in Moller) while TMAC actually has an opposite profile in J2731 and E2586. Why did the authors focus on this gene? It would be good to sort genes into different profiles and then systematically compare them with the reported phenotypes.*

*Response:* We appreciate the reviewer’s thoughtful suggestion and agree that a systematic classification of different transcriptional response profiles could provide additional insight. Our gene selection approach was guided by the hypothesis that HKT1-mediated Na⁺ transport influences lateral root development under salt stress by modulating transcriptional networks, particularly those involved in ABA signaling.

To identify candidate genes, we initially focused on genes that exhibited conserved regulation patterns in both backgrounds in response to salt stress and HKT1 overexpression, as these may represent core mechanisms underlying lateral root inhibition. However, we also examined genes with genotype-specific expression patterns, as differences between Col-0 (E2586) and C24 (J2731) may reflect background-dependent regulatory mechanisms.

TMAC2 was prioritized for further investigation because it emerged as a key regulator of ABA signaling, a known mediator of lateral root development under salt stress. While TMAC2 expression was upregulated in E2586 but downregulated in J2731 under HKT1 overexpression, this pattern aligns with the idea that genetic background influences the interaction between Na⁺ transport and ABA signaling, leading to ecotype-specific transcriptional responses. Instead of being an anomaly, this differential expression provided an opportunity to investigate how HKT1 may influence ABA homeostasis in different genetic contexts.

We have now revised the manuscript to better explain our gene selection criteria and ensure that the rationale behind our focus on TMAC2 is clear. Additionally, we recognize the value of categorizing genes based on their expression patterns across backgrounds and have improved our discussion to highlight distinct transcriptional profiles and their potential links to lateral root inhibition.

*Reviewer #2 comment: The Discussion could be better focussed. Hypothetical statements, especially not linked to the genes investigated should be omitted.*

*Response:* We appreciate the reviewer’s feedback and have revised the Discussion to improve focus and clarity. Specifically, we have removed hypothetical statements that were not directly linked to the genes investigated and refined our conclusions to remain closely aligned with the experimental data.

Additionally, we have ensured that our discussion maintains a clear narrative by emphasizing the key findings related to HKT1, TMAC2, and TIP2;2, while providing a well-supported interpretation of their roles in lateral root inhibition under salt stress. We believe these revisions make the discussion more precise and directly relevant to the study’s central research question.

*Reviewer #2 comment: I struggle to understand what exactly is being concluded. Yes, clearly gene functions and responses differ in the two genetic backgrounds, which is probably not too surprising but what does this mean? Do the transcriptomics results help to pin down the differences? How many SNPs between Col-0 and C24???? Could it be that the previously reported different root phenotypes in the HKT1 overexpression lines are not due to differences in HKT1 localisation but to other differences in the two backgrounds?*

*Response:* We appreciate the reviewer’s request for greater clarity regarding the conclusions drawn from our findings. To address this, we have revised the Discussion to more explicitly state what our results reveal about the relationship between HKT1, TMAC2, TIP2;2, and lateral root inhibition under salt stress, and how genetic background influences these responses.

We acknowledge that differences in gene function and response across genetic backgrounds are not unexpected, but our study provides transcriptomic evidence that helps pinpoint specific regulatory differences between Col-0 and C24. We have now clarified how these differences manifest at the transcriptional level and how they relate to the previously observed variations in lateral root phenotypes between HKT1 overexpression lines.

Regarding the number of SNPs between Col-0 and C24, while we recognize that natural variation could contribute to the observed differences, a comprehensive SNP analysis is beyond the scope of this study. However, we have revised the manuscript to acknowledge this possibility and highlight the need for future studies to determine whether HKT1-dependent phenotypic differences are primarily driven by variation in HKT1 expression domains or by broader genetic differences between Col-0 and C24.

*Reviewer #2 comment: The authors try to explain their observations with ‘feedback loops’ and ‘complex regulatory networks’. A scheme depicting at least a working hypothesis would certainly help the reader.*

*Response:* We thank the reviewer for this suggestion. We have now added the figure (Fig. 9) depicting the regulatory complex network that is based on the results presented in this study.

*Reviewer #3 comment: This is a very competent study of genes associated with the reduction of lateral root growth by salt stress. In particular it looks at the role of HKT1 or TIP2;2 overexpression lines in modifying the response patterns. As one would expect, the responses are complex. The responses are described well, but I could not find a hypothesis or the outline of a coherent narrative that the results would (or not) ‘fit’. This made the significance of the work difficult for me to fully understand.*

*Response:* We thank the reviewer for their constructive and thoughtful comments. As in responses to other reviewers - we have attempted to improve articulation of our hypothesis within this manuscript. *Reviewer #3 comment: The focus of the paper is not clear. Is the central topic the mechanism of reduction of lateral roots under salt stress? And to what extent this inhibition is explained by increased sensitively to ABA due to expression of TMAC2 and/or OX of HKT1? If so, this comparatively straightforward, simple aim is not stated clearly. There seems to be ambivalence as to whether inhibition of lateral root growth is caused by increased ABA (line 97) or by increased Na+ in the pericycle (line 105).*

*Response:* We appreciate the reviewer’s request for greater clarity regarding the focus of the paper. To address this, we have explicitly stated the central research question in the Introduction and refined the framing of our hypothesis throughout the manuscript. The primary focus of our study is to investigate the mechanism by which HKT1-mediated Na⁺ transport influences lateral root development under salt stress, with a particular emphasis on the role of ABA signaling.

We have also clarified how TMAC2 contributes to ABA accumulation and lateral root inhibition, and how HKT1 overexpression further modulates these responses in a genetic background-dependent manner. The revised manuscript now better distinguishes whether lateral root inhibition is primarily driven by increased ABA signaling or by Na⁺ accumulation in the pericycle. While our results suggest that both factors contribute to root system remodeling, the transcriptomic and genetic evidence supports a model in which HKT1 and TMAC2 interact within an ABA-dependent regulatory network that influences lateral root growth.

To eliminate any ambiguity, we have revised the relevant sections to ensure that our findings are presented as a coherent mechanism, rather than as competing explanations. This should now make it clear how ABA sensitivity, Na⁺ accumulation, and genetic background collectively shape lateral root responses to salt stress. *Reviewer #3 comment: How this all gels with function of TIP2;2 is not clear. A simple diagram of the connections between root growth mechanisms and salt tolerance is needed.*

*Response:* We thank the reviewer for this suggestion. We have now added the figure (Fig. 9) depicting the regulatory complex network that is based on the results presented in this study.

*Reviewer #3 comment: Lastly, I suggest that there should be information presented at the start of the paper, on the reason to compare the Arabidopsis ecotypes Col-0 and C24, and how they might differ in Ca signaling pathways or in whatever might explain a different result of the overexpression of HKT1. The paper should include a discussion of the results in relation to the genomic or transcriptomic differences of the two ecotypes. Just saying “genetic background” is not sufficient.*

*Response:* We thank the reviewer for this valuable suggestion. To address this, we have added a clearer explanation in the Introduction and the initial Results section regarding the rationale for comparing Col-0 and C24. Specifically, we now highlight how these backgrounds differ in their HKT1 expression domains, genetic variation, and potential regulatory differences that could influence stress responses.

Additionally, we have now added Fig. S6, where we show that there is very little allelic variation in TMAC2 promoter between Col-0 and C24. Moreover, as we see opposite effect of TMAC2 overexpression in both backgrounds, this genomic context dependency of the TMAC2 - HKT1 - ABA response is more complicated than we set out to explore within this manuscript.

We have extensively revised the Discussion to provide a more detailed interpretation of how the observed transcriptomic differences between Col-0 and C24 relate to lateral root inhibition under salt stress. While we acknowledge that genetic background alone is a broad term, we have now clarified how specific transcriptomic responses help pinpoint regulatory differences contributing to the distinct phenotypes in the two backgrounds, and that the main explanations for this response are likely found downstream of TMAC2.

A full genomic comparison between Col-0 and C24, including potential differences in Ca²⁺ signaling pathways or other stress-related regulatory networks, is beyond the scope of this manuscript. However, we now acknowledge the need for future studies to explore whether the differential phenotypic effects of HKT1 overexpression stem primarily from its tissue-specific localization or from broader genomic variations between the two ecotypes.
